# Exploring the vitamin biosynthesis landscape of the human gut microbiota

**DOI:** 10.1128/msystems.00929-24

**Published:** 2024-09-17

**Authors:** Chiara Tarracchini, Gabriele Andrea Lugli, Leonardo Mancabelli, Douwe van Sinderen, Francesca Turroni, Marco Ventura, Christian Milani

**Affiliations:** 1 Laboratory of Probiogenomics, Department of Chemistry, Life Sciences, and Environmental Sustainability, University of Parma, Parma, Italy; 2 Microbiome Research Hub, University of Parma, Parma, Italy; 3 Department of Medicine and Surgery, University of Parma, Parma, Italy; 4 APC Microbiome Institute and School of Microbiology, Bioscience Institute, National University of Ireland, Cork, Ireland; University of California San Diego, La Jolla, California, USA

**Keywords:** bacteria, micronutrients, microbiome

## Abstract

The human gut microbiota possesses the capacity to synthesize vitamins, especially B group vitamins, which are recognized as indispensable for various biological processes both among members of these bacterial communities and host cells. Accordingly, vitamin production by intestinal commensals has attracted significant interest. Nevertheless, our current understanding of bacterial vitamin synthesis is primarily based on individual genomic and monoculture investigations, therefore not providing an overall view of the biosynthetic potential of complex microbial communities. In the current study, we utilized over 100 bacterial genes known to be involved in the biosynthesis of B group and K vitamins to assess the corresponding vitamin biosynthetic potential of approximately 8,000 human gut microbiomes. Our analyses reveal that host-associated factors, such as age and geographical origin, appear to influence the diversity and abundance of vitamin biosynthetic pathways. Furthermore, we identify gut microbiota members that substantially contribute to these biosynthetic functions at each stage of human life. Interestingly, inference of microbial co-associations and network relationships uncovered the apparent key role played by folate and cobalamin in equilibrium establishment of the infant and adult gut microbial communities, respectively.

**IMPORTANCE:**

Overall, this study expands our understanding of microbe-mediated vitamin biosynthesis in the human gut and may provide potential novel targets to improve availability of these essential micronutrients in the host.

## INTRODUCTION

The human gastrointestinal tract is colonized by a multitude of microorganisms, known as the gut microbiota, with up to 10^12^ bacterial cells per gram of intestinal content, which are believed to interact with the host at the mucosal surface (1, 2). In this inter-kingdom interactive relationship, the fitness of the overall microbe–host structure, the so-called holobiont, relies on a variegated set of molecular exchanges between the symbiotic partners, which may involve small bioactive molecules with major physiological impact (3–5) and may include short-chain fatty acids, indole lactic acids, and (metabolic intermediates of) vitamins. Specifically, the latter represent micronutrients that generally act as biosynthetic precursors of essential cofactors used in a myriad of metabolic and regulatory processes, being indispensable for both host and the microbial communities it harbors (6, 7). Although humans are incapable of synthesizing most vitamins, various enteric bacterial species have been shown to synthesize vitamin K (menaquinones), as well as most of the water-soluble B vitamins, such as thiamine (B1), riboflavin (B2), niacin (B3), pantothenic acid (B5), pyridoxine (B6), biotin (B7), folate (B9), and cobalamin (B12) (8–12). Interestingly, recent studies demonstrating the presence of B vitamins in bacterial cell culture media, while also providing evidence for distinct vitamin uptake systems in Gram-positive and Gram-negative bacteria, have supported the notion that these microbe-derived micronutrients may, under certain circumstances, be released extracellularly, possibly through cell lysis, thereby facilitating cross-feeding within microbial communities (9, 13–16).

However, because a significant portion of the microorganisms residing in the human intestine has not been cultivated to date, experimental validation and quantification of vitamin production by each human gut commensal has been a major challenge. In this context, alternative approaches not involving cultivation, such as shotgun metagenomic DNA sequencing, metatranscriptomics, and metabolomics, represent powerful tools to allow prediction of the biodiversity and function of complex microbial communities. Recently, it has been estimated that more than a quarter of the Dietary Reference Intake (DRI) of four B vitamins may be provided by the human gut microbiota (12). Although such a calculation may be highly conjectural, it supports the notion that gut-associated microbial communities can effectively contribute to host vitamin status (17, 18). Notably, although dietary vitamins are reported to be mainly absorbed in the small intestine, the human endothelial cells of the large intestine also contain transporters for various vitamins (17, 18), such as FOLR1 for folate (19), RFT1 for riboflavin (20), as well as the multivitamin transporters SMCT1 (21) and SMVT (22), emphasizing the host’s commitment to acquire (microbial-derived) vitamins.

In the current study, microbial genes predicted to be involved in vitamin biosynthetic pathways were explored in more than 8,000 human gut metagenomes, enabling assessment of the vitamin biosynthetic potential of the gut microbiota across the human lifespan and various geographical origins. Moreover, by assigning species-level taxonomical classification to microbial genes predicted to be involved in vitamin pathways, we reveal prominent microbial commensals that may contribute to vitamin production in the human gut microbiome.

## MATERIALS AND METHODS

### Collection of metagenomic data from the human gut microbiome

In this study, 8,076 cross-sectional shotgun metagenomic sequencing data from 3,340 infants aged 0–3 years, 4,091 adults (18–70 years old), and 645 elderly individuals (>70 years old), that had been recruited from different geographical areas, using Illumina technology were retrieved from the Sequence Read Archive (SRA) database. A complete list of samples is available in the supplementary material (Table S1). According to the associated metadata, all metagenomic samples analyzed in this study are from individuals who were overall healthy, had no history or clinical evidence of gastrointestinal diseases, and were not taking antibiotics at the time of sample collection. To capture the complete microbial biodiversity of the healthy human gut, no additional selection based on population characteristics, such as type of birth, sex, BMI, or diet was performed while retrieving the metagenomic data set. However, dietary information was collected from the metadata associated with the metagenomic data sets where available. To ensure sufficient sequencing data, samples that did not reach the threshold of 5 million reads, enabling the reliable, functional assessment of human stool metagenomes (23), were discarded. Moreover, to avoid potential confounding effects of library size, abundance data were normalized based on the total number of reads in each metagenomic sample.

### Processing of the metagenomic data set and identification of microbial vitamin-related gene sequences

The collected shotgun sequencing data were filtered for quality (minimum mean quality score, 20; window size, 5 bp; and minimum length, 100 bp) using the fastq-mcf program, and metagenomic reads aligning/mapping to the *Homo sapiens* genome sequence were identified through Blastn program and removed. Association of the filtered metagenomic reads with metabolic functions was performed using an implemented function of the software METAnnotatorX2 (24). Specifically, the metagenomic data sets were screened against the MetaCyc metabolic database composed of 18,973 metabolites to identify all attributable enzymatic processes. The Enzyme Commission (EC) numbers were assigned to nucleotide sequences having 80% of minimum query cover (e-value cutoff 1e-10) using the Diamond utility program integrated into the METAnnotatorX2 pipeline. Selection of microbial genes corresponding to enzymes (EC numbers) involved in vitamin biosynthesis was identified after a comprehensive literature search (Table S2).

Beta diversity of compositional differences in microbial genes involved in microbial vitamin biosynthesis was evaluated using QIIME2 (qiime diversity core-metrics-phylogenetic) through pairwise permutational analysis of variance (PERMANOVA) statistics based on Bray–Curtis indices. Principal coordinate analysis (PCoA) plots for beta diversity metrics were generated using ORIGIN 2021 (https://www.originlab.com/2021).

To identify the primary microbial vitamin producers in each metagenomic fecal sample, the metagenomic reads corresponding to genes involved in each vitamin biosynthetic pathway were pooled and then taxonomically classified with the software METAnnotatorX2 (24). The taxonomic abundances were normalized to generate compositional data, and the median abundance was used as a measure of central tendency for such data.

### Co-occurrence network analysis

Covariance analysis involving the bacterial species obtained by taxonomic profiling of vitamin-related genes was realized employing Kendall’s tau rank covariance analysis. The obtained correlation coefficients were parsed using the Gephi software (https://gephi.org/) to build a force-driven network whose nodes represent bacterial species, and edges define their relationships. The node size is related to the magnitude of contribution in folate (Fig. 4d) and cobalamin (Fig. 4e) biosynthetic pathways, whereas the edge color shows the type of interaction, i.e., positive (green) or negative (red).

### Statistical analyses

The software SPSS version 25 and OriginPro graphing and analysis 2021 (www.ibm.com/software/it/analytics/spss/) (https://www.originlab.com/) were used for statistical tests and graphing unless otherwise specified. The sample size was estimated based on previous experience and sample availability. Differences in the abundance of microbial genes involved in vitamin biosynthetic pathways were tested by Kruskall Wallis test with Dunn’s *post hoc* test and Bonferroni multiple hypothesis corrections. Differences in the abundance of microbial taxa were tested through Mann–Whitney U test, and the Benjamini–Hochberg method was applied to control the false discovery rate at the *P*-value threshold of 0.05. To investigate the differences in the microbial vitamin-related gene patterns, compositional data were converted to a distance matrix using the Bray–Curtis distance indices utilizing the Vegan R package. The hierarchical clustering analysis (HCA) was performed on the obtained Bray–Curtis matrix using Pearson correlation as a distance metric and the sum square of distances and furthest neighbor for the clustering method. The optimal number of clusters was defined through a silhouette analysis. Permutational analysis of variance (PERMANOVA) and analysis of similarities (ANOSIM) were conducted through QIIME 2 software with 999 random permutations on the same Bray–Curtis matrix.

## RESULTS AND DISCUSSION

### Collection of the metagenomic shotgun data set covering the human lifespan

A total of 8,076 international, publicly available shotgun metagenomic fecal samples from 3,340 infants, 4,091 adults, and 645 elderly individuals, all defined as healthy based on their corresponding metadata, were collected and clustered according to age to investigate the gut microbiome-associated vitamin biosynthetic potential across the lifetime (Table S1). Specifically, and similar to what was previously described (25), age groups were named I (infants, 0–3 years of age), MA (middle-aged adults, 18–70 years old), OA (old adults, 71–79 years old), and E (elderly being 80 years and older). Moreover, to evaluate the impact of environmental factors, such as diet and lifestyle, on the abundance and diversity of microbial-mediated vitamin metabolic pathways within each age category, we used metagenomic data sets of individuals from geographical areas with marked lifestyle differences (26, 27). Accordingly, based on available metadata, we selected 692 metagenomic fecal samples from the easternmost regions of the world, such as China, North/South Korea, and Japan, 698 samples from Italy, and 1,573 samples from the USA (associated with a western lifestyle and an ultra-processed food diet) (28).

### The abundance of gut microbiota-associated vitamin biosynthesis genes depends on host age

As mentioned above, vitamins can be classified as small organic molecules with a pronounced biological impact (4, 29). Previously, we reported substantial age-associated differences in the overall production of microbial bioactive small metabolites (4). Here, we further explored developmental and aging functional trajectories of the human gut microbiome, focusing on microbial genes involved in vitamin biosynthetic pathways. For this purpose, the metagenomic sequenced reads were classified based on Enzyme Commission (EC) categories using the MetaCyc as reference database, identifying 103 essential enzymes involved in the biosynthesis of eight water-soluble B group vitamins and the fat-soluble vitamin K (in the form of menaquinone, vitamin K2) (Table S2).

**Fig 1:**
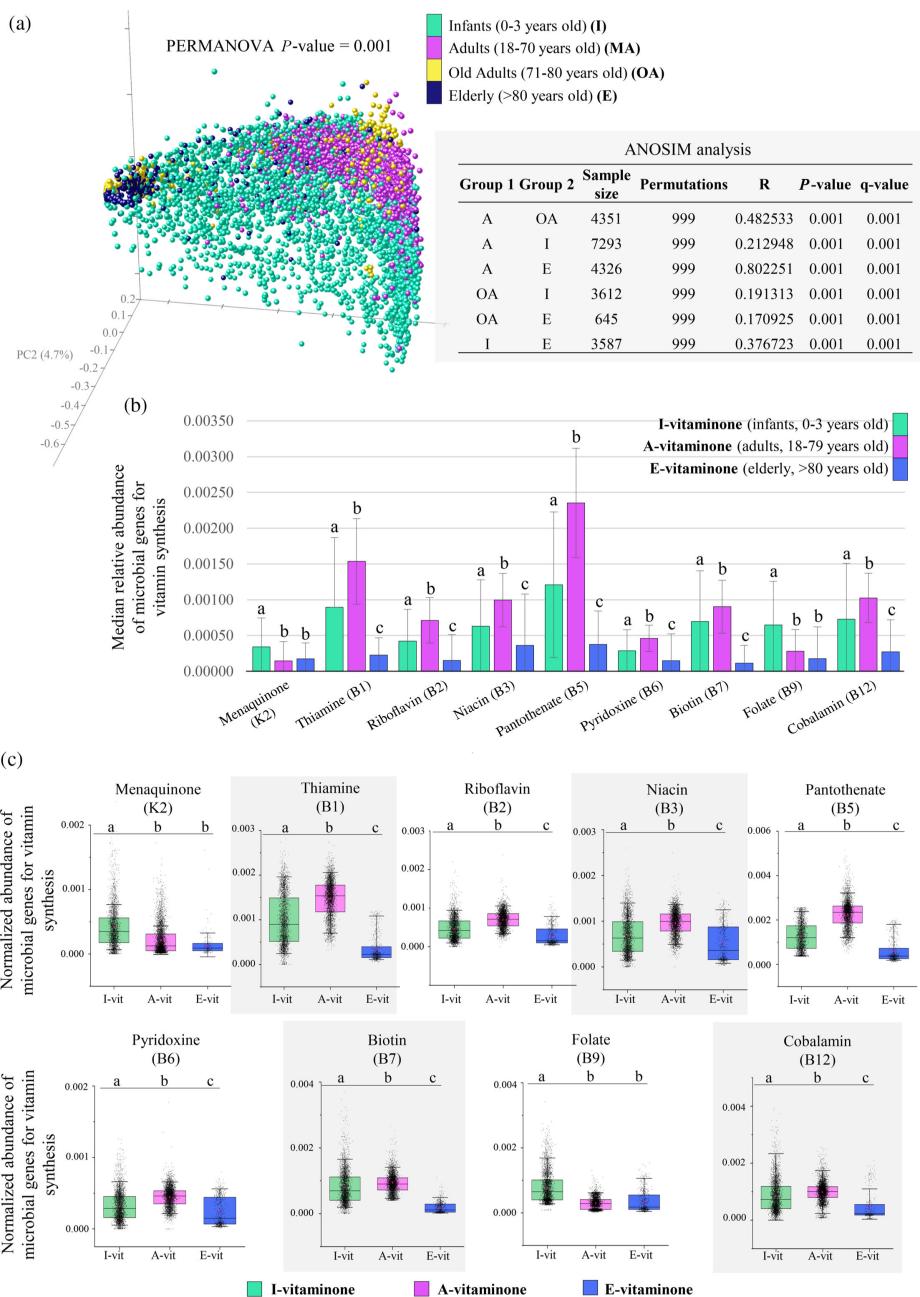
Microbiota-derived vitamin biosynthetic genes within fecal metagenomes across the human lifespan. Age-based differences in the distribution and abundance of microbial vitamin biosynthetic pathways were depicted through the principal coordinate analysis (**a**), bar chart (**b**), and box and whisker plot (**c**). In (**b**), error bars are determined by the interquartile range (IQR), representing the difference between the third quartile (Q3) and first quartile (Q1) of the data set. This measure of dispersion is less affected by the presence of outliers compared with standard deviation. The boxes are determined by the 25th and 75th percentiles, and the whiskers are determined by the 5th and 95th percentiles (**c**). Each age group showed a particular pattern of microbe-associated vitamin biosynthetic genes, leading to the definition of the I-vitaminome, A-vitaminome, and E-vitaminome, being typical of the infant, adult, and elderly gut microbiota, respectively. The lowercase letters show statistical significance: different letters indicated statistically significant differences (Kruskal–Wallis test with Dunn’s *post hoc* test, Bonferroni-adjusted *P* < 0.05). In contrast, groups sharing the same lowercase letter are statistically similar (Kruskal–Wallis test with Dunn’s *post hoc* test, Bonferroni-adjusted *P* > 0.05).

Examination of vitamin-associated gene abundance within fecal metagenomes across the human lifespan revealed discernible discrepancies that are closely associated with host age (PERMANOVA *P* = 0.001) (Fig. 1a). Specifically, the most marked differences in microbial vitamin biosynthetic pathways were identified between the adult population and healthy elderly aged 80 years and older (ANOSIM, R = 0.80 at *P* = 0.001; PERMANOVA, pseudo F = 2,303.4 at *P* = 0.001) (Fig. 1a; Fig. S1; Table S3). Indeed, as shown in Fig. 1a, deduced vitamin synthetic abilities of the gut microbiota of older individuals under 80 years were still relatively analogous to those of younger adults (ANOSIM, R = 0.48 at *P* = 0.001; PERMANOVA pseudo F = 868.2 at *P* = 0.001) (Fig. 1a; Fig. S1; Table S3). In contrast, above the age of 80 years, the biosynthetic microbial ability for seven of the nine vitamins examined was shown to undergo a substantial reduction (Kruskal–Wallis test with Dunn’s *post hoc* test, Bonferroni-adjusted *P* < 0.0001) (Fig. 1a), reflecting the continuous and progressive biological decline that affects not only the elderly physiology but also the functionality of its intestinal microbiota (30).

As a result of these observations, the entire set of gut microbiota-harbored vitamin biosynthetic genes, here defined as the vitaminome, was classified according to host age, with MA and OA groups merged, leading to the identification of the I-vitaminome (Infants, 0–3 years old), A-vitaminome (Adults, 18–79 years old), and the E-vitaminome (Elderly, >80 years old) (Fig. 1b; Table S3). Specifically, a comparative analysis reveals that the most represented biosynthetic pathways of the A-vitaminome were those for thiamine (B1) and pantothenate (B5), followed by niacin (B3) and biotin (B7), whereas menaquinone (vitamin K2), pyridoxine (B6), and folate (B9) were relatively underrepresented (Fig. 1b; Table S3).

Compared with healthy adults, the infant population showed a reduction in abundance ranging from 21% to 49% across six of the nine microbial vitamin biosynthetic pathways (Fig. 1b, Table S3). Although this finding mirrors a functionally incomplete microbiota maturation within the first 3 years following birth, the biosynthesis pattern of the I-vitaminome remained rather overlapping with the A-vitaminome (ANOSIM, R = 0.21 at *P* = 0.001; PERMANOVA pseudo F = 905.2 at *P* = 0.001) (Fig. 1a; Fig. S1; Table S3), reflecting a situation in which an adult-like microbiota composition begins to establish in the infant’s intestine at the end of the first year of life (31, 32). However, compared with both the A- and E-vitaminome, the I-vitaminome was characterized by a significantly higher abundance of genes for the biosynthesis of folate and menaquinones (Kruskal–Wallis test with Dunn’s *post hoc* test, Bonferroni-adjusted *P* < 0.0001) (Fig. 1b and c; Table S3), demonstrating a robust ability of the infants’ gut microbial communities to synthesize these essential vitamins. Moreover, we previously observed a higher abundance of microbial biosynthetic pathways for folate in the gut microbiota of breastfed infants compared with those receiving formula (4), suggesting a close link between folate biosynthetic potential, early dietary patterns, and specific infant gut pioneers, including well-documented bifidobacterial species known to proliferate in the gut of infants fed with breastmilk (33–35).

### Vitamin biosynthetic potential of the human gut microbiota across geographical locations

Environmental variables encompassing habitat types, diet, cultural tradition, and lifestyle, strongly influence gut microbiota composition and function, resulting in country-specific microbial signatures (26, 27, 36, 37). To inspect the effect of these factors on the biosynthetic abilities of gut-derived vitamins in the adult population, i.e., A-vitaminone, we compared the gut-derived biosynthetic potential of age-matched healthy individuals living in distinctly separate geographical locations, including metropolitan areas in the USA (*n* = 366), the easternmost regions of the world (*n* = 368), and the Italian peninsula (*n* = 438) (Table S1).

**Fig 2:**
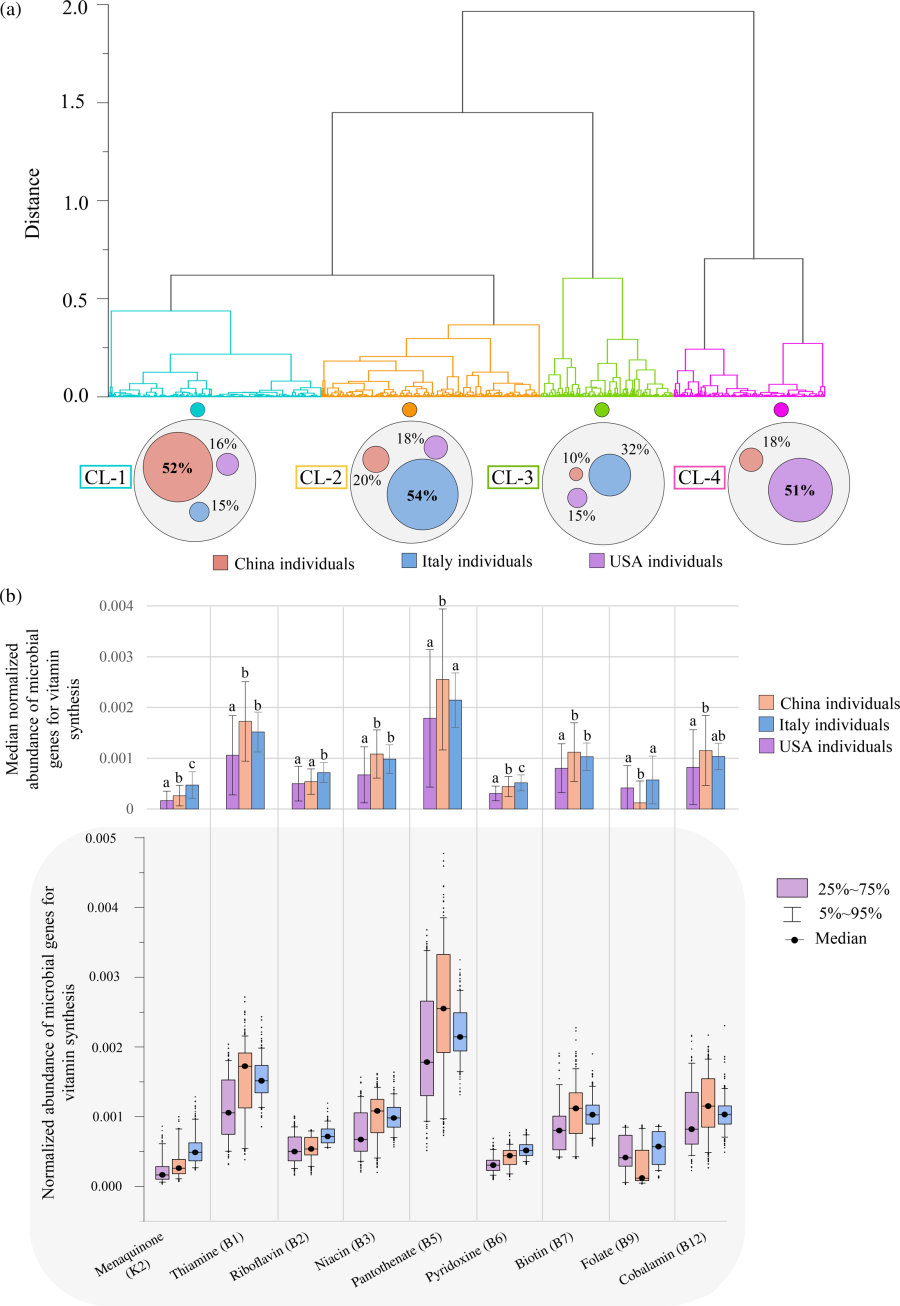
Patterns of vitamin biosynthetic potential of the human fecal microbiota across geographical areas. In panel (a), Hierarchical cluster analysis (HCL) revealed three distinct clustering patterns (CL-1, –2, and −4) of microbe-associated vitamin biosynthetic genes based on the geographical origin of 772 metagenomic samples. In panel (**b**), variations in the abundance of biosynthetic genes across fecal metagenomes from distinct geographical locations were visualized for each assessed vitamin pathway, utilizing bar chart and box-and-whisker plot. In the bar chart, error bars are determined by the interquartile range (IQR), representing the difference between the third quartile (Q3) and first quartile (Q1) of the data set. This measure of dispersion is less affected by the presence of outliers compared with standard deviation. In the box-and-whisker plot, boxes are determined by the 25th and 75th percentiles, and the 5th and 95th percentiles determine whiskers. The lowercase letters show statistical significance: different letters indicated statistically significant differences (Kruskal–Wallis test with Dunn’s *post hoc* test, Bonferroni-adjusted *P* < 0.05). In contrast, groups sharing the same lowercase letter are statistically similar (Kruskal–Wallis test with Dunn’s *post hoc* test, Bonferroni-adjusted *P* > 0.05).

A silhouette clustering method was utilized to analyze recurring patterns of microbial vitamin-related genes across the metagenomic data sets, unveiling four robust clusters named CL1–CL4 (Fig. 2a). Interestingly, more than 50% of each population was assigned to a specific cluster (Fig. 2a), disclosing a distribution of vitamin-synthesizing genes that markedly correlate with the host’s geographical origin.

Upon closely examining microbial vitamin-related biosynthesis pathways present in the cohorts from three distinct geographic areas, we observed a noteworthy similarity in the abundance of biosynthesis genes for half of the investigated vitamins between the populations of China and Italy (Kruskal–Wallis test with Dunn’s *post hoc* test, Bonferroni-adjusted *P* > 0.05) (Fig. 2b; Table S4). However, healthy Italian individuals showed a significantly higher abundance of microbial-derived biosynthetic pathways for menaquinone (K2), pyridoxine (B6), folate (B9), and riboflavin (B12) compared with Chinese or US populations (Kruskal–Wallis test with Dunn’s *post hoc* test, Bonferroni-adjusted *P* < 0.0001), whereas gut microbiomes from China showed a significantly enriched presence of genes involved in pantothenate (B5) biosynthesis (Kruskal–Wallis test with Dunn’s *post hoc* test, Bonferroni-adjusted *P* < 0.0001) (Fig. 2b; Table S4). Conversely, the gut microbiota of US individuals seemed to be less genetically committed to producing vitamins. Indeed, on average, the abundance of microbe-derived genes for vitamin biosynthesis was reduced in the gut microbiota of the US adult population by 19%–51% when compared with that of the age-matched Italian and Chinese cohorts (Fig. 2b; Table S4), supporting the idea that lifestyle and a diet rich in processed foods typical of highly westernized and industrialized areas may be associated with a significant reduction in the abundance of gut microbiota-derived vitamin biosynthetic pathways (38, 39).

By contrast, vitamins whose biosynthetic genes were enriched in the Italian population exert a marked gastroprotection role because of their antioxidant, anti-aging, and anti-inflammatory effects (40–44). Nevertheless, by considering the effect of key dietary components (plant-rich vs meat-rich diets) on the bacterial B vitamin-related gene profiles, we found that the Italian cohort can be further divided into two compact sub-populations according to dietary habits (IT-hP and IT-hM; Fig. S2a and b). However, a small portion (13%) of Italian individuals with a plant-rich diet falls within the meat-rich diet cluster (Fig. S2a). This could be due to potential bias in reporting dietary habits that may generate outliers despite the rigor of the methodology, or the actual influence of additional different factors, such as other dietary components not considered in this study. Otherwise, some individuals may have modified their diet or related elements shortly before enrollment, leading to an evolving gut microbiota that may still reflect the composition associated with their previous dietary habits.

More in detail, our investigation on a subset of the Italian population suggests that a plant-rich diet with reduced meat intake tends to enhance the abundance and diversity of B vitamin-producing genes, particularly for vitamins B2, B6, and K2 (Mann–Whitney U test, Bonferroni-adjusted *P* < 0.0001) (Fig. S2c; Table S5). Conversely, a meat-rich diet appears to significantly reduce the abundance of gut microbiota-derived genes responsible for vitamin B12 biosynthesis (Mann–Whitney U test, Bonferroni-adjusted *P* < 0.05) (Fig. S2c; Table S5). This outcome may be due to the fact that dietary cobalamin and its derivates, abundant in animal-derived foods, can escape absorption in the small intestine, thereby selecting B12-utilizing bacteria in the colon. Meanwhile, high-fiber diets typically favor the growth of butyrate-producing bacteria, such as *Faecalibacterium prausnitzii*, as well as members of the *Prevotella* and *Ruminococcus* genera, which are associated with increased diversity and abundance of microbial genes responsible for vitamin synthesis (45, 46).

Although dietary habits significantly impact vitamin-associated functions of the gut microbiota within the same geographical population, variation in the abundance of vitamin-related microbial genes was still observable to a lesser extent between populations sharing similar plant-based diets from different geographical areas (Italy vs. USA) (Mann–Whitney U test, Bonferroni-adjusted *P* < 0.01) (Table S5; Fig. S2d and e). This likely reflects the influence of additional country-specific factors beyond single diet components. Indeed, environmental exposures, local dietary supplements or medications, and lifestyle habits that are difficult to standardize or quantify can act in concert with the genetic background of the host population to modulate the human gut microbiota composition and activity, including its overall vitamin synthesis potential.

The impact of geographical origins was also evaluated within the I- and E-vitaminone (Tables S6 and S7). Interestingly, the gut microbiome-derived vitamin biosynthetic potential of elderly individuals from easternmost regions displayed a global depletion in vitamin biosynthetic pathways, ranging from approximately 8% to 70% compared with other countries, across eight of the nine vitamins investigated (Kruskal–Wallis test with Dunn’s post *hoc test*, Bonferroni-adjusted *P* < 0.001) (Table S6). Although these data does not directly imply a disadvantaged health condition of individuals from eastern regions, it may reflect a distinct composition of the gut microbiota, likely correlated with variations in diet, lifestyle, and country-specific healthcare interventions.

In contrast, geographic origin did not appear to substantially affect the vitamin biosynthetic potential of the infant gut microbiome (Table S7). Indeed, only biosynthetic genes for cobalamin, menaquinone, and pantothenate were differentially abundant across different geographical locations. Specifically, although microbe-derived pantothenate biosynthetic pathway was more abundant in Chinese and Italian infants, those for cobalamin and menaquinone exhibited higher levels in the US infant population (Kruskal–Wallis test with Dunn’s *post hoc* test, Bonferroni-adjusted *P* < 0.001) (Table S7). In this context, country/ethnicity-specific composition of maternal milk and available infant formulas, weaning practices and timing, as well as perinatal care may have contributed to generating variations in vitamin-related gut microbiome functionalities in the infant population worldwide.

### Phylogenetic origin of vitamin biosynthetic genes in the human gut microbiota

**Fig 3:**
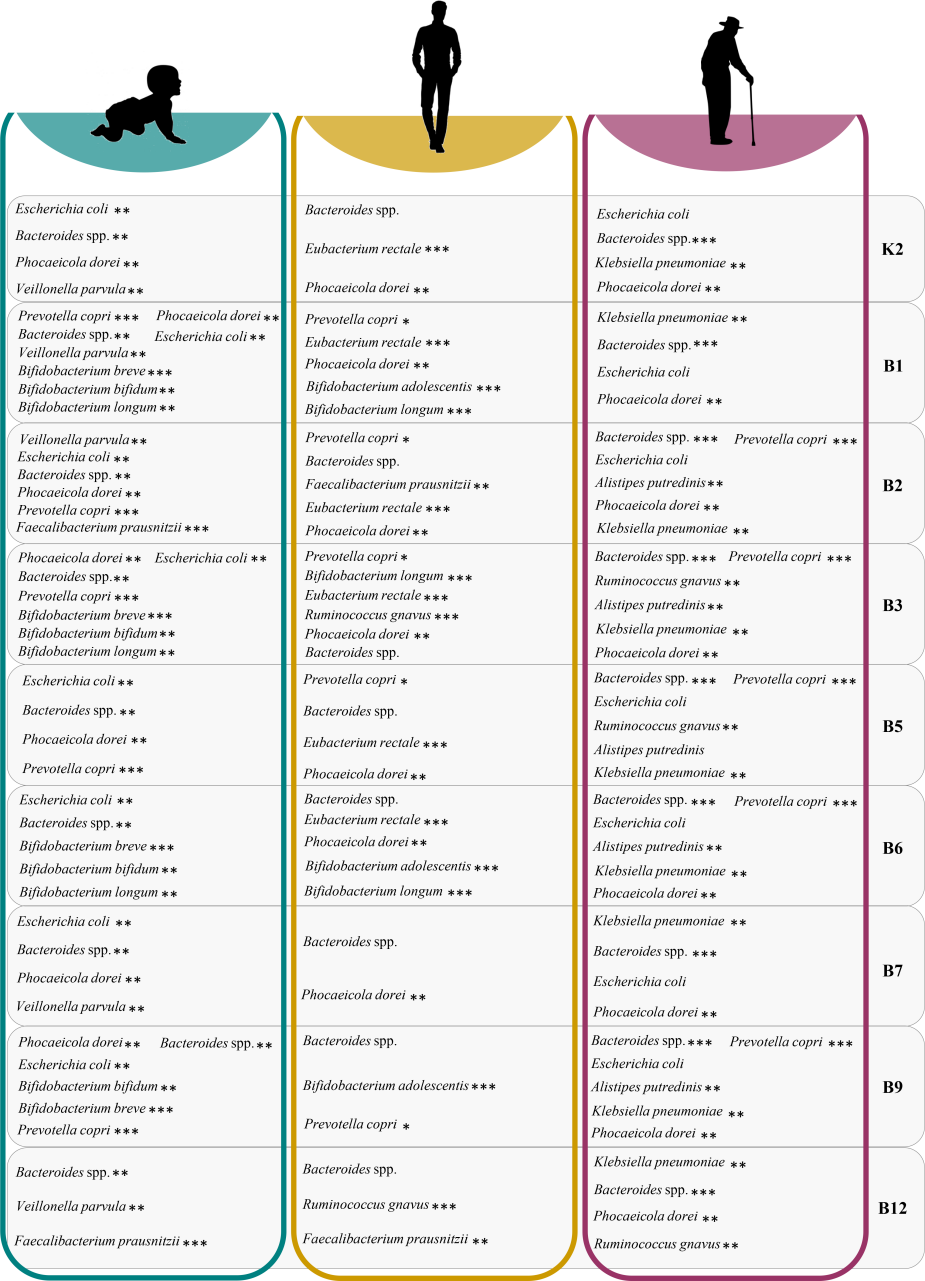
Key microbial contributors to age-related vitamin biosynthetic pathways encoded by the gut microbiota. For each host age stage, the top 10 intestinal microbiota members contributing to the predicted vitamin biosynthetic pathways are reported for every screened vitamin. For each specific vitamin, only bacterial species contributing more than 2% to the biosynthetic pathways were included in the report. The number of asterisks corresponds to the number of pairwise comparisons in which the abundance of the given bacterial species was significantly different when the age group was further subdivided based on geographic origin (China, Italy, and the USA). This can provide an insight into the extent of variation in the major vitamin-producing species based on geographical location.

To further explore the vitamin biosynthetic capabilities of the human gut microbiome, the metagenomic reads assigned to vitamin biosynthesis within each age-associated vitaminome were subjected to taxonomic classification, obtaining profiles of microbial taxa predicted to be involved in each vitamin biosynthetic pathway (Fig. 3; Tables S8 to S10). To assess the validity of our metagenomic-derived expectations, we compared our predictions with existing experimental data and studies from available scientific literature evaluating the genomic presence of complete vitamin biosynthetic pathways (Table S11). Specifically, when we considered microbial species predicted to contribute significantly to vitamin biosynthetic pathways (median relative abundance of vitamin-associated metagenomic reads >5% at least for one vitamin), we found that more than 90% of our predictions were consistent with genomic assessments or experimental evidence of B-vitamin production (Table S11). This slight inconsistency can likely be due either to the challenging functional assignment of short reads for species with lower metagenomic relative abundance or the genomic divergences often observed among strains of the same species. This latter is the case for *F. prausnitzii* and *B. longum* subsp. *infantis* taxa, whose riboflavin production has been demonstrated *in vitro* only for specific strains (16, 47). Overall, the examined data from literature are in good agreement with our *in silico* prediction of B-vitamin biosynthetic abilities (Table S11).

**Fig 4:**
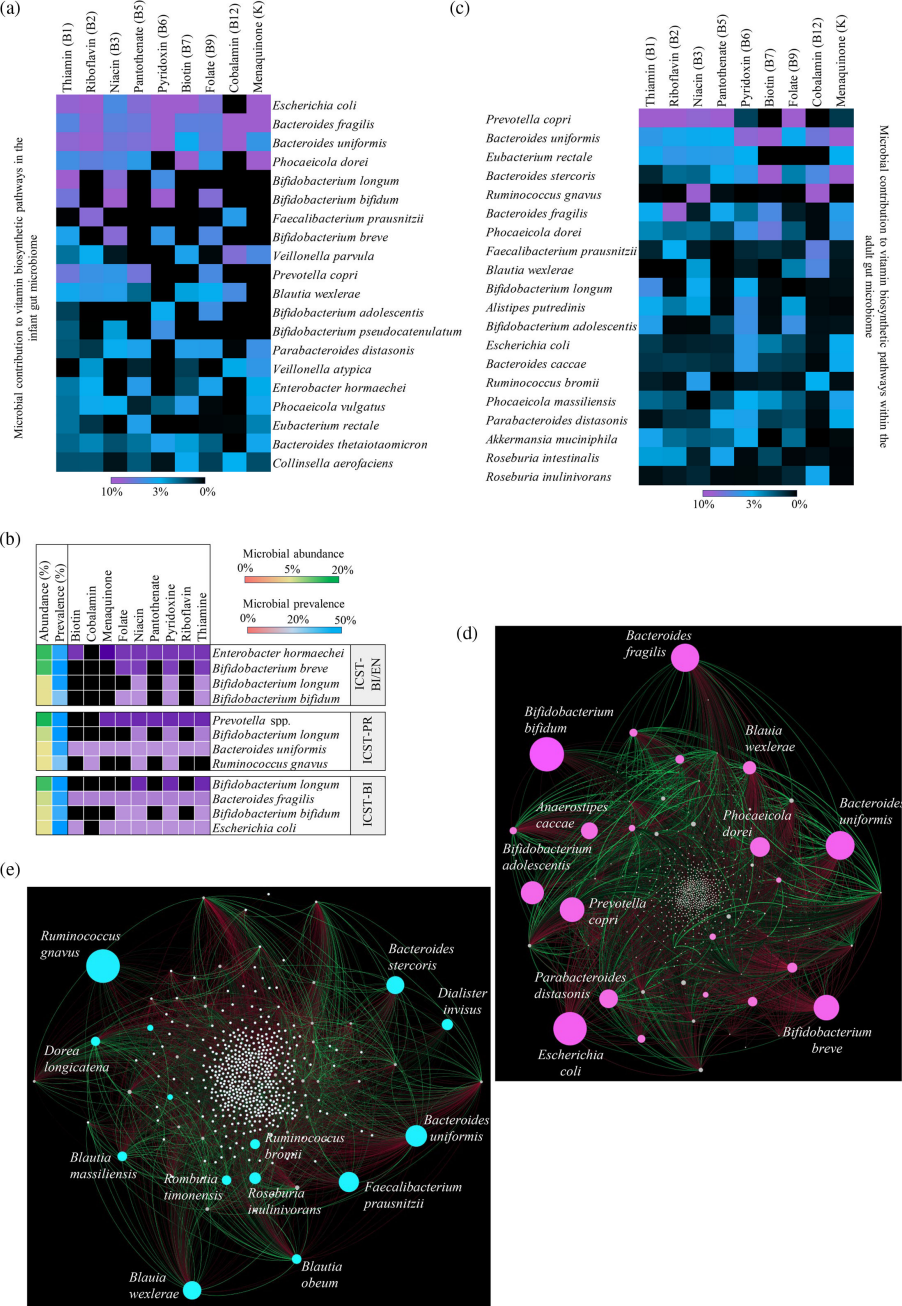
Co-occurrence patterns of human gut microbiota members identified as main contributors to vitamin biosynthetic potential. Heatmaps illustrate relative contributions of key members in the infant (**a**) and adult (**c**) gut microbiota to vitamin biosynthesis, determined through taxonomic classification of vitamin-related gene sequences. Panel (**b**) shows the association between vitamin biosynthetic potential and previously identified healthy community state types (CSTs) of the infant gut microbiota (48). Violet cells represent the presence of vitamin biosynthetic pathways, and the color intensity correlates with the abundance of bacterial species identified within the ICST. Panels (**d**) and (**e**) represent the microbial interaction networks among members of the infant and adult gut microbiota, respectively. Each node represents a bacterial species, and the size is proportional to the amount of the predicted contribution to folate (**d**) and cobalamin (**e**) biosynthesis within the infant (**d**) and adult (**e**) gut microbiota, obtained from taxonomic profiling of vitamin biosynthetic genes. Edge width is proportional to the significance of supporting evidence, and different colors indicate the sign of the association (green positive, red negative).

**Fig 5:**
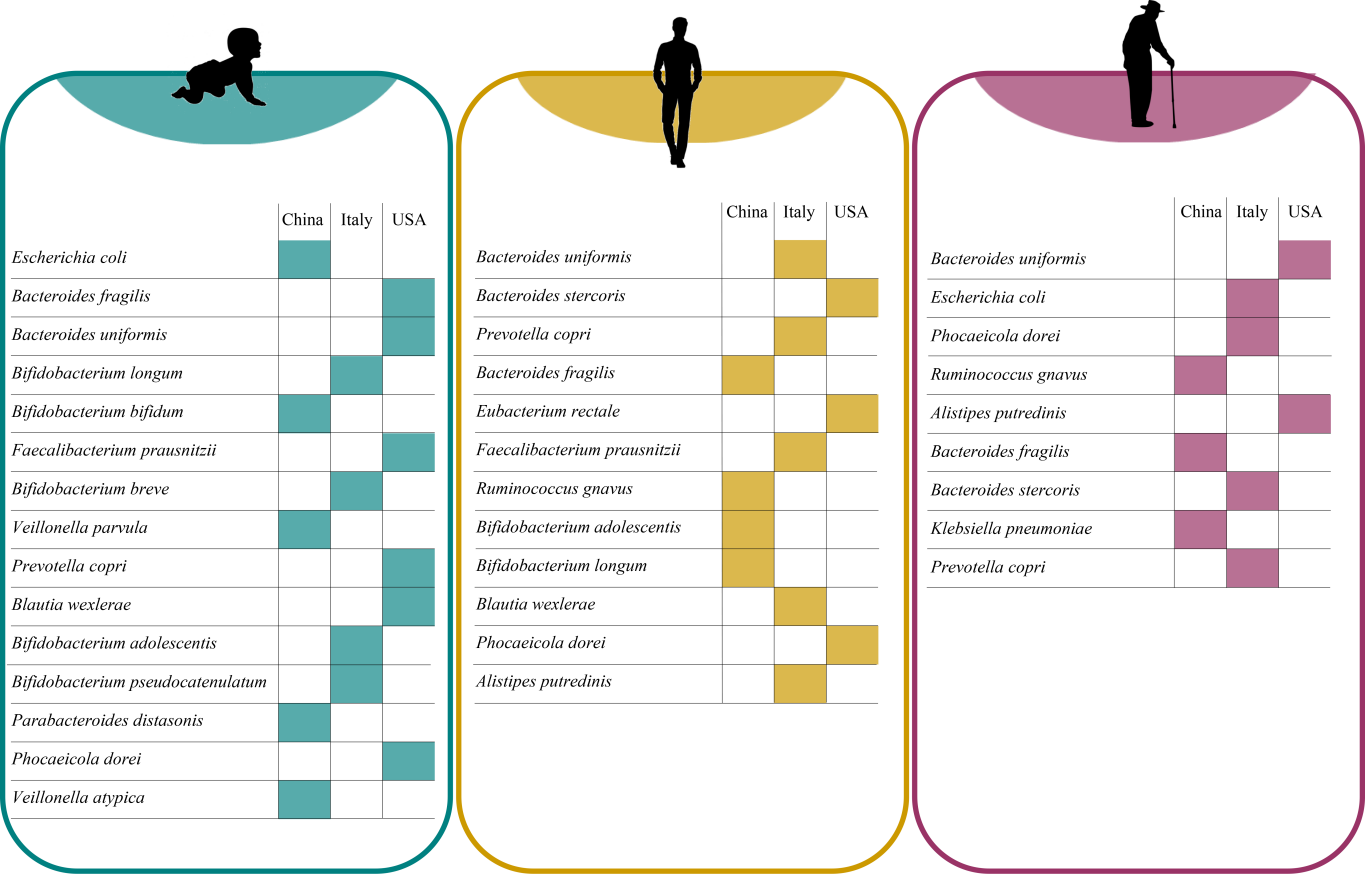
Variation in the abundance of the main vitamin-producing bacteria based on geographical origin. Within each age group**,** the average abundance of the key vitamin-producing species was calculated according to the geographical origin (China, Italy, and USA as proxy of dietary habits). For a given bacterial species, colored cells indicate which geographical location exhibited the maximum average abundance among the three considered. Statistical significance of the comparison between countries was calculated by the Kruskal–Wallis with Dunn’s *post hoc* test reported in Tables S8 to S10.

Within the I-vitaminome, the metagenomic reads related to B-vitamins and menaquinone biosynthesis were mainly derived from *Escherichia coli*, constituting median relative abundances ranging from 6.20% to 20.97% of the entire bacterial population predicted to participate in vitamin biosynthesis (Fig. 4a; Table S8). Similarly, also members of the *Bacteroides* genus appeared to contribute significantly to all nine inspected vitamin pathways (median relative abundance from 3.54% to 18.67%), whereas *Bifidobacterium* species were associated with the genetic arsenal for the biosynthesis of folate, niacin, pyridoxine (up to median relative abundance of 9.77%, 10.60%, and 10.30%, respectively, from the key producer *Bifidobacterium bifidum*), and thiamine (up to median abundance of 11.93% from the key producer *Bifidobacterium longum*) (Fig. 4a; Table S8). Interestingly, upon considering various geographical origins (USA, Italy, and China), we observed a significant enrichment of microbial species belonging to the *Bacteroides* genus in the gut microbiomes of US infants compared with other locations (Kruskal–Wallis test with Dunn’s post *hoc test*, Bonferroni-adjusted *P* < 0.05) (Fig. 5; Table S8). This finding may account for the relatively higher proportions of biosynthetic genes for cobalamin and menaquinone described above in this population (Table S7). Meanwhile, infants from Italy and China were more abundant in members of the *Bifidobacterium* genus and *E. coli*, which collectively provide the genetic biosynthetic potential for eight of the nine vitamins investigated (Kruskal–Wallis test with Dunn’s post *hoc test*, Bonferroni-adjusted *P* < 0.05) (Fig. 5; Table S8).

Based on the observed distribution of vitamin biosynthetic potential among members of the infant gut microbiota, we exploited the previously identified infant community state types (ICSTs) (48) to link healthy-associated microbial communities to putative microbial vitamin biosynthetic patterns. Specifically, considering the more abundant ICST members (up to the average relative abundance of 5%), it appears that the vitamin biosynthetic potential of the ICST-BI/EN, predominantly composed of bifidobacterial species, may be primarily confined to four essential B-group vitamins, possibly reflecting the reduced biodiversity in microbial genera (Fig. 4b).

By contrast, the ICST-PR and ICST-BI constituted by *Bifidobacterium* and *Bacteroides* species, along with *Prevotella* genus members or *E. coli* respectively, appeared to have the potential to produce a broader spectrum of vitamins, indicating that there may be variations in vitamin biosynthetic abilities among different healthy states of the infant gut microbial communities (Fig. 4b). Moreover, several dominant early gut colonizers, including members of the *Bacteroides* genus, *E. coli*, and *B. bifidum,* convey genes for the *de novo* biosynthesis of folate and menaquinone, correlating with the higher relative abundance of biosynthetic pathways for these vitamins in infants compared with adults as described above.

In a similar fashion, the A-vitaminome was also examined, leading to the definition of *Bacteroides* species, *P. copri*, and *E. rectale*, as the predominant species for wide-ranging B-vitamin biosynthesis, followed by *F. prausnitzii* and *Ruminococcus gnavus*, which contributed exclusively to the biosynthesis of cobalamin (B12), niacin (B3), and riboflavin (B2) (Fig. 4c; Table S9). Interestingly, although *Bifidobacterium* species were less abundant in the adult gut microbiomes compared with those of infants (average relative abundance of 3.69% ± 0.18% vs 22.29 ± 0.28%), *B. longum*, and *Bifidobacterium adolescentis* persist in contributing to the genetic arsenal for folate, niacin, pyridoxine, and thiamine biosynthesis in adulthood, with median relative abundances ranging from 2.70% to 5.00% (Fig. 4c; Table S9), possibly emphasizing the life-long functional relevance of bifidobacteria to the health of human physiology and metabolism (49–51).

In contrast, consistent with the compositional remodeling of the gut microbiota occurring with advancing age (52), the vitamin-related genes in healthy elderly population were assigned to members of the *Enterobacteriaceae* family, including *Klebsiella pneumoniae* (median relative abundance from 1.40% to 5.97% across all the nine vitamins), and *E. coli* (median relative abundance up to 8.36%) (Table S10). Despite that, from our analysis, species within the *Bacteroides* genus emerged as the most prominent for synthesizing a diverse array of B vitamins from infancy to old age (Fig. 3; Tables S8 to S10). Indeed, the relatively large genomes of these microbial species were predicted to contain genes for the *de novo* synthesis of all eight B vitamins, including cobalamin, which is typically *de novo* synthesized only by a minority of gut-associated bacteria.

Despite common age-specific signatures, assessing the impact of different geographical locations on the abundance of the main microbe contributors to the A-vitaminone revealed that only seven of the 43 statistically differential abundant major vitamin-producing species (Kruskal–Wallis test *P* < 0.05) were enriched in US individuals compared with other countries (Fig. 5; Table S9), which may at least in part to explain the extensive depletion of the vitamin genetic potential observed above for this population. Conversely, individuals from Italy and China exhibited a higher abundance of *B. uniformis*, and *P. copri*, or members of the *Bifidobacterium* genus and *R. gnavus*, respectively (Kruskal–Wallis test with Dunn’s post *hoc test*, Bonferroni-adjusted *P* < 0.05) (Fig. 5; Table S9). Similarly, geographic location particularly impacted the abundance of *P. copri* and *Bacteroides* genus in the elderly population, with a marked reduction in older individuals from China (Kruskal–Wallis test with Dunn’s post *hoc test*, Bonferroni-adjusted *P* < 0.05) (Fig. 5; Table S10), correlating with a broad decline in vitamin biosynthetic pathways described above for this cohort.

Interestingly, from these findings, it emerged that geographic differences, likely arising from the multifaceted interactions of lifestyle factors affecting gut microbiota composition, are reflected in the microbial genetic potential for vitamin biosynthesis in an age-specific manner.

Overall, our metagenome-based predictions confirm and extend at the whole-community scale what was previously suggested at individual genome level (12). Specifically, our findings unveiled the species-specific contribution of human gut commensals to the vitamin biosynthetic potential of the whole microbial communities, ultimately enabling the identification of the major age-specific vitamin-producing bacteria. Moreover, by considering different geographical locations, i.e., China, USA, and Italy, as a proxy for dietary preferences and lifestyle habits, our findings highlighted country-specific variation of bacterial species associated with vitamin biosynthetic potential.

### Co-occurrence and network analysis of gut microbiota members suggest vitamin metabolism sharing

Complex bacterial communities, such as the human gut microbiota, are shaped by the combined effects of both positive and negative microbial interactions (13). Among these, trophic webs of shared metabolic features represent a crucial ecological force generating and maintaining cohesion among members of such microbial consortia (53, 54). Vitamins are frequently implicated in cross-feeding events (6, 8, 16, 55), as they are generally essential for enzymatic function in bacterial cells and are energetically expensive to synthesize. Although the reason why microbes release costly compounds in quantities sufficient to support the growth of other species might not be immediately apparent, accidental excretion, occurring due to cell lysis, has been proposed as one of the mechanisms explaining such bacterial cross-feeding events (56, 57).

Accordingly, contingent upon possessing specific transporters for uptake, vitamin auxotroph bacteria can exploit the presence of prototrophic partners to proliferate, therefore establishing solid associations that contribute to microbial community assembly (56, 58–60). Based on these considerations, we performed co-occurrence analyses among adult and infant gut commensals. Specifically, we examined the interconnections among the primary contributors to different vitamin synthetic pathways identified above, using the Spearman correlation coefficient as an indicator of the direction and strength of their relationships.

Notably, within the infant gut microbiota, *Veillonella parvula* emerged as one of the most significant microbial partners of *E. coli* (Spearman correlation, *ρ* = 0.42, *P* = 7.48^−12^) (Table S12). Intriguingly, *V. parvula* exhibits limited vitamin biosynthetic potential compared with *E. coli* (Fig. 4a; Table S8), therefore possibly relying on the latter for its B vitamin requirements. On the other hand, *V. parvula* possesses genetic capabilities for cobalamin biosynthesis, a trait absent in *E. coli* (Fig. 4a; Table S8). Although various and complex interconnected factors may contribute to shaping microbial relationships, these findings, along with the identification of a specific uptake transporter for vitamin B12 in *E. coli* (61), suggest sharing of micronutrients between these gut microbiota members.

Similarly, several *Bifidobacterium* species, including *B. bifidum*, *B. longum*, and *B. breve*, are significantly correlated with *Enterobacter hormaechei* (Spearman correlation, *ρ* coefficient ranging from 0.23 to 0.40, all *P* = 0) (Table S12), having also been recently identified as the primary bacterial species constituting one of the healthy-associated infant enterotypes (Fig. 4b) (48). In this co-association, although growth of bifidobacterial species is supported by menaquinones (62, 63)*, E. hormaechei* appeared able to produce vitamin K2 (Fig. 4a; Table S8), thus possibly facilitating the access of bifidobacteria to environmental vitamin K2.

Intriguingly, besides the single microbe–microbe interplays described above, the reconstruction of a global co-occurrence network highlights that folate biosynthesis represents an important force in driving microbe interactions within the infant gut. Indeed, members of folate-producing *Bifidobacterium* and *Bacteroides* genera, along with *E. coli*, emerged as central nodes of the infant gut microbial community (Fig. 4d, pink circles), orchestrating the dynamics of microbe–microbe interactions.

Similar observations can be drawn from the gut microbiota of adults. Notably, *Bacteroides uniformis*, predicted to have the potential to synthesize all B vitamins and menaquinone (vitamin K), exhibited significant association with *Alistipes putredinis*, *B. adolescentis,* and *F. prausnitzii,* (Spearman correlation, *ρ* coefficient of 0.51, 0.26, and 0.25, respectively, all *P* = 0), whose biosynthetic abilities were predicted to be limited to a B vitamin subset (Fig. 4c; Table S13). In addition, also *Prevotella copri* showed a significant affinity to various *Bacteroides* species (Spearman correlation, *ρ* coefficient from 0.37 to 0.46, *P* = 0) Table S8(Table S13), coinciding with possible syntrophic vitamin biosynthetic pathways (Fig. 4c). This phenomenon is notably conceivable for cobalamin (vitamin B12). Indeed, the disentanglement of network structure within the adult gut microbiome disclosed prominent hubs corresponding to cobalamin-producing bacteria, guiding the interaction network of the whole gut microbe community (Fig. 4e). This is consistent with the limited prevalence of the *de novo* cobalamin biosynthesis pathway among gut microbiota members due to its well-known complexity (more than 20 genes) and energetically costly biosynthesis (64, 65), which may generate phenomena of symbiosis and cooperation, as well as competition for environmental resources of this vitamin (65–67). Indeed, by metabolic reconstruction, although the ability for B12 biosynthesis has been predicted for approximately 40% of human gut-associated bacteria, a substantial majority, ranging from 60% to 80%, exhibit auxotrophy (12, 14, 68, 69).

Overall, these findings suggest that members of the human gut microbiota with a high rate of co-association may exhibit opposite and complementary vitamin auxotrophies and prototrophies, resulting in a collectively expanded vitamin biosynthetic ability (16, 70).

Moreover, although genomic and metagenomic *in silico* investigations display inherent limitations in predicting bacterial metabolic potentials, our findings provide valuable insights to guide *in vitro* experiments needed to confirm possible vitamin-dependent (syn)trophic relationships among human gut microbiome members.

### Conclusions

Previous research has comprehensively addressed the production of B-group vitamins by members of the human gut microbiota, primarily by comparing the genome contents of various well-known microorganisms inhabiting the human gut (12, 16). However, despite making significant contributions to elucidating conserved capacities and species-specific peculiarities related to B vitamin biosynthesis across human gut commensals, this research relied on reference genomes excluded from their ecological context (niche).

In the current study, extending beyond previous genome-level assessments, we investigated the metagenomic-derived vitamin biosynthetic pathways of the human gut microbiome, highlighting the impact of various host-associated factors on the prevalence and abundance of microbial vitamin biosynthetic pathways. Specifically, the developmental stage of host physiology demonstrated a remarkable ability to discriminate patterns of gut microbe-derived vitamin biosynthetic potential, leading to the identification of the I-vitaminome, A-vitaminome, and the E-vitaminome as the genetic potential for vitamin production specific to infant, adult, and elderly gut microbiome, respectively. Our analyses, coupled with taxonomic profiling of vitamin biosynthetic pathway-associated reads, revealed that pathways for folate (B9) and menaquinone (K2) syntheses were more abundant in early life than in adulthood, reflecting the dominance of the gut microbiota by *Bifidobacterium* genus and *E. coli* . Similarly, within the adult population, the genus *Bacteroides* not only has the potential to produce all eight B vitamins and menaquinones, as previously suggested (12, 70), but also appears to be the major contributor in terms of the relative abundance of vitamin biosynthetic genes within the entire gut microbial community, followed by *P. copri*, and *Eubacterium rectale*.

Moreover, our findings suggest that geographical origin, likely reflecting differences in diet and lifestyle, exerts a predictable impact on the microbial vitamin biosynthetic potential. In particular, we observed a noteworthy association between being an adult of USA origin and a substantial, widespread reduction in the abundance of B-vitamin biosynthetic pathways derived from the gut microbiome. Notwithstanding, in our *in silico* findings, we found that individuals following similar plant-based diets from different geographical locations continued to show differences in the profiles of vitamin-related genes, indicating that dietary habits are only one component of geographic location-specific elements shaping the biosynthetic potential for vitamins in the human gut microbiota. Accordingly, additional factors, likely influenced by geographic and cultural contexts, such as environmental elements, lifestyle practices, and even genetic backgrounds may contribute to these observed differences.

It is worth acknowledging that, despite its numerous advantages, metagenomic predictions are constrained by certain limitations. Foremost among these is the inability to discern which genes or pathways are actively transcribed into functional proteins. Consequently, although this aspect lies beyond the scope of our present investigation, it remains plausible that the expression of genes implicated in *de novo* microbial vitamin biosynthesis may be governed by regulatory mechanisms not considered herein. In this context, our *in silico* predictions hold the potential to inform further experimental studies aimed at validating and elucidating the production, as well as the intricate regulatory mechanisms, governing microbial vitamin biosynthesis.

Increasing evidence has shown that microbe-derived vitamins can be released into the environment through cell lysis during growth (13, 71, 72), and the human large intestine can absorb microbially synthesized water-soluble vitamins through specialized transport systems (29, 73, 74). Therefore, although it seems more likely that, in a complete and adequate dietary regimen, the primary source of B vitamins for the host is the diet, it has been proposed that vitamins derived from elements of the gut microbiota not only influence the intestinal environment, being available to intestinal human cells and immune system functions, but may also play a minor yet notable role in contributing to the host’s B vitamin status (12, 17, 18, 75).

Nevertheless, the precise contribution of microbially synthesized vitamins to host physiology remains yet to be elucidated. To the best of our knowledge, only one study has attempted to assess the extent of this contribution, highlighting that gut microbiota-derived vitamins potentially contribute approximately 27% to 86% of the reference intakes for vitamins B2, B6, B9, and B12 in the adult population (12). Despite their commendable qualities, these approximations did not account for bacterial utilization. It is reasonable to assume that a large portion of the vitamins produced in the colon is consumed by enteric microorganisms to support their own metabolism and growth, including other non-vitamin-producing bacteria, likely reducing the vitamin pool available to the host compared with the predictions. Indeed, due to their energetically demanding biosynthesis, vitamins are frequently subject to recycling, competition or symbiotic cross-feeding within microbial communities, leading to intricate microbe–microbe interactions (56, 70, 76). Consistent with this notion, our results revealed a significant co-occurrence of microbial species exhibiting complementary potential for vitamin production. This observation suggests that the well-known complex trophic webs shaping the human gut microbiota plausibly entail the sharing and community-wide exchange of vitamins, metabolic intermediates of vitamins, or other vitamin-related products. Specifically, our findings from microbial network inference highlight that the potential to produce folate and cobalamin represent central functions underpinning inter-microbe relationships within the infant and adult gut microbiota, respectively. Consequently, deviations from the optimal composition and functionality of the gut microbial community, as observed in various human diseases, may result in impaired vitamin biosynthesis (77). This eventuality may disrupt the equilibrium of the whole microbial community, with repercussions on host physiology beyond the gastrointestinal environment.

In this context, better understanding of vitamin production by gut commensals and the microbe–microbe connections it generates has the potential to open avenues for developing strategies to influence and optimize these symbiotic relationships (78, 79).

## SUPPLEMENTAL MATERIAL

Figure S1 msystems.00929-24-s0001.pdf Analysis of similarities of gut microbiome-derived vitamin biosynthetic patterns.

Figure S2 msystems.00929-24-s0002.pdf Variation in vitamin-biosynthetic genes based on dietary preferences within and between human populations.

Supplemental Tables msystems.00929-24-s0003.xlsx Tables S1 to S13.

ASM does not own the copyrights to Supplemental Material that may be linked to, or accessed through, an article. The authors have granted ASM a non-exclusive, world-wide license to publish the Supplemental Material files. Please contact the corresponding author directly for reuse.
