# Supplementary material for: Exploring the vitamin biosynthesis landscape of the human gut microbiota
Source: mSystems. 2024 Sep 17;9(10):e00929-24. doi: 10.1128/msystems.00929-24 (PMC11494892; doi:10.1128/msystems.00929-24)
Supplement: Figure S1 — Analysis of similarities of gut microbiome-derived vitamin biosynthetic patterns. [file msystems.00929-24-s0001.pdf]

a)

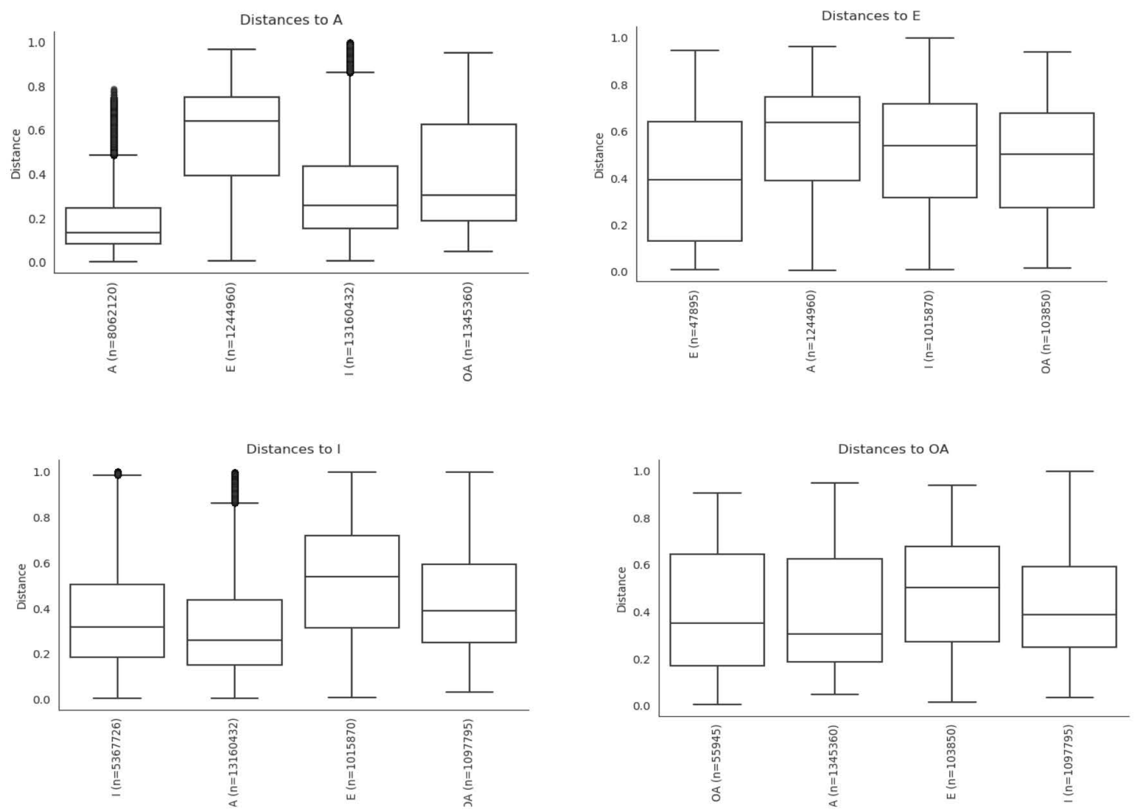

b)

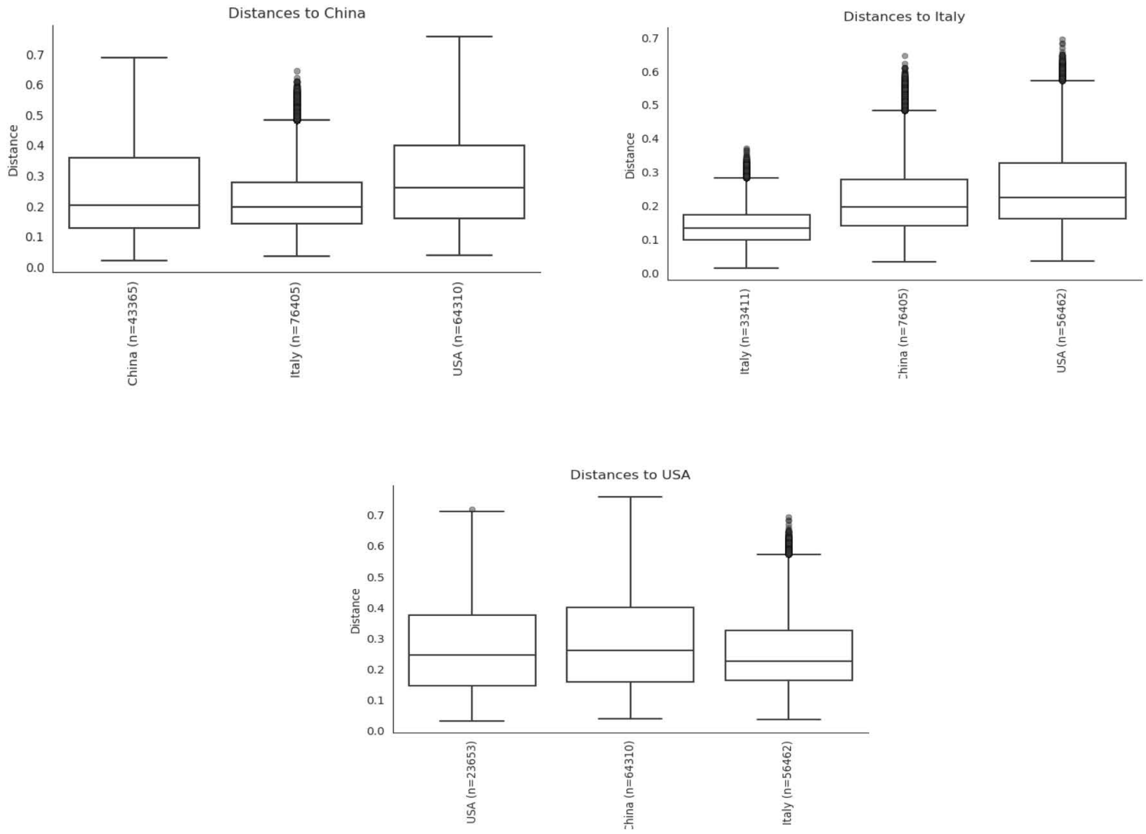

Figure S1. Analysis of similarities (ANOSIM) of gut microbiome-derived vitamin biosynthetic patterns. Panel (a) illustrates intergroup distances in vitamin biosynthetic patterns following stratification of the population based on age (A= Middle-Aged adults, 18-70 years; OA=older adults, 71-79 years; E=elderly, >80 years; I=infants, 0-3 years). Panel (b) displays the same comparison among human populations from different geographical areas.
