# Supplementary material for: Exploring the vitamin biosynthesis landscape of the human gut microbiota
Source: mSystems. 2024 Sep 17;9(10):e00929-24. doi: 10.1128/msystems.00929-24 (PMC11494892; doi:10.1128/msystems.00929-24)
Supplement: Figure S2 — Variation in vitamin-biosynthetic genes based on dietary preferences within and between human populations. [file msystems.00929-24-s0002.pdf]

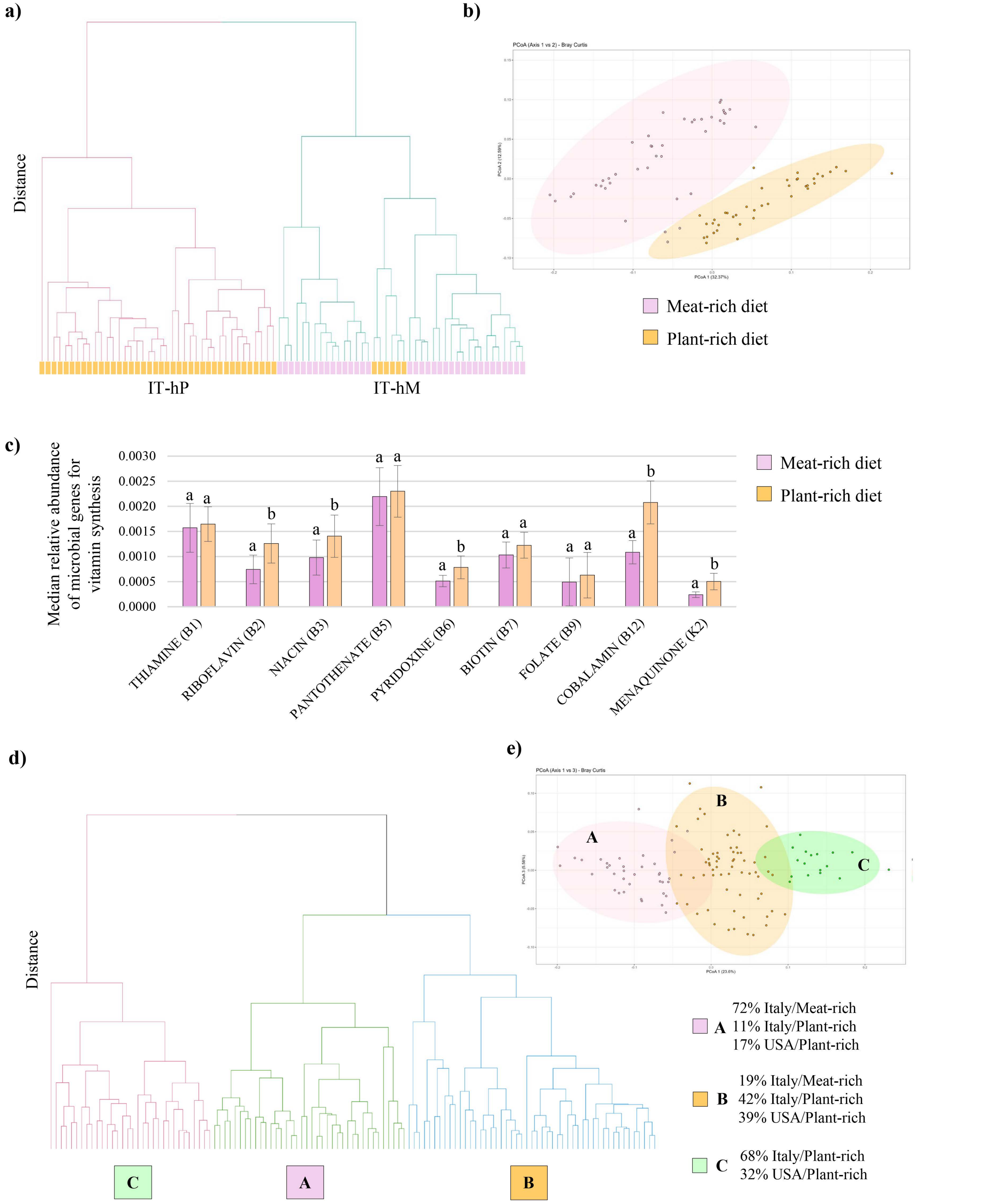

Figure S2. Variation in vitamin-biosynthetic genes based on dietary preferences within and between human populations. Differences in diversity and abundance of microbial vitamin biosynthetic pathways between Italian individuals following a plant-rich diet or meat-rich diet were depicted through Hierarchical Clustering Analysis **(a)**, Principal Coordinate Analysis **(b),** and bar chart **(c)**. In **(c)**, error bars are determined by the Interquartile Range (IQR), representing the difference between the third quartile (Q3) and the first quartile (Q1) of the dataset. The lowercase letters show statistical significance: different letters indicated statistically significant differences (Mann-Whitney U Test, Bonferroni-adjusted *p*-values < 0.05). On the contrary, groups sharing the same lowercase letter are statistically similar (Mann-Whitney U Test, Bonferroni-adjusted *p*-values < 0.05).

Similar analyses were performed including individuals from USA with a plant-based diet. Panel **(d)** and panel **(e)** depict the Hierarchical Clustering Analysis and Principal Coordinate Analysis, respectively, of individuals from Italy (meat- and plant-rich diet) and USA (plan-riched diet). The proportion of country- and diet-specific categories within each cluster is indicated by percentages.
